# Identification of novel risk factors for postoperative severe hypocalcemia in patients with primary hyperparathyroidism undergoing parathyroidectomy: a case control study

**DOI:** 10.1186/s12902-024-01620-6

**Published:** 2024-06-12

**Authors:** Jiahao Xu, Na Kong, Nan Bai, Ziqin Zhang, Aimin Cui, Shen Tan, Qiqi Xu

**Affiliations:** General Surgery of Beijing Jishuitan Hospital, Capital Medical University, The Fourth Clinical Medical College of Peking University, 68 Huinanbei Road, Changping District, Beijing, 100096 China

**Keywords:** Primary hyperparathyroidism, Parathyroidectomy, Postoperative severe hypocalcemia, Hungry bone syndrome, Serum phosphorus, Osteocalcin, Calcium supplementation

## Abstract

**Background:**

Patients with primary hyperparathyroidism (PHPT) are at risk for severe hypocalcemia (SH) following parathyroidectomy (PTX), but limited data exist on the predictors of SH. We aimed to identify risk factors for early postoperative SH after PTX in patients with PHPT and to evaluate the predictive value of clinical parameters.

**Methods:**

A retrospective review of patients with PHPT who underwent PTX between January 2010 and December 2022 was performed. A total of 46 patients were included in the study, with 15 (32.6%) experiencing postoperative SH, 19 (41.3%) having calculi in the ureter or kidney, and 37 (80.4%) having osteoporosis. Patients were divided into SH and non-SH groups based on postoperative serum calcium levels. Preoperative biochemical indicators, bone turnover markers, and renal function parameters were analyzed and correlated with postoperative SH.

**Results:**

Statistically significant (*P *< 0.05) differences were found in preoperative serum calcium (serum Ca), intact parathyroid hormone, serum phosphorus (serum P), serum Ca/P, percentage decrease of serum Ca, total procollagen type 1 intact N-terminal propeptide, osteocalcin (OC), and alkaline phosphatase levels between the two groups. Multivariate analysis showed that serum *P* (odds ratio [OR] = 0.989; 95% confidence interval [95% CI] = 0.981–0.996; *P* = 0.003), serum Ca (OR = 0.007; 95% CI = 0.001–0.415; *P* = 0.017), serum Ca/P (OR = 0.135; 95% CI = 0.019–0.947; *P* = 0.044) and OC levels (OR = 1.012; 95% CI = 1.001–1.024; *P* = 0.036) were predictors of early postoperative SH. The receiver operating characteristic curve analysis revealed that serum *P* (area under the curve [AUC] = 0.859, *P* < 0.001), serum Ca/P (AUC = 0.735, *P* = 0.010) and OC (AUC = 0.729, *P* = 0.013) had high sensitivity and specificity.

**Conclusion:**

Preoperative serum P, serum Ca/P and osteocalcin levels may identify patients with PHPT at risk for early postoperative SH after PTX.

## Introduction

Primary hyperparathyroidism (PHPT) is a prevalent endocrine disorder that is characterized by the hypersecretion of parathyroid hormone (PTH) by the parathyroid glands. Its pathological etiology is commonly a solitary benign parathyroid adenoma. This disease manifests with diverse clinical features, including bone lesions, calculi, abdominal and neurological symptoms, as well as involvement of various organ systems such as kidneys, muscles, cardiovascular system, and nerves. In rare cases, a parathyroid crisis may occur [1]. The definitive treatment for PHPT is parathyroidectomy (PTX), which aims to achieve surgical resection of the offending adenoma and attain a biochemical cure for PHPT [2].

Complications following PTX may include recurrent laryngeal nerve injury, symptomatic hematoma, permanent hypoparathyroidism, and postoperative hypocalcemia [3, 4]. Hypocalcemia is the most frequently observed complication after PTX, with the serum calcium (serum Ca) level typically reaching its lowest level within 24–48 h post-surgery. Common symptoms of hypocalcemia include paresthesia, muscle spasms, cramps, tetany, circumoral numbness, and seizures [5]. Physical examination of individuals with hypocalcemia may elicit the Chvostek's and Trousseau's signs. Prolonged hypocalcemia but normal PTH levels after surgery for PHPT are referred to as ‘hungry bone syndrome’. High percentage of hypocalcemia referred to the hungry bone syndrome is found typically in patients with severe PHPT and preoperative high bone turnover or in renal hyperparathyroidism [5].

Hypocalcemia can be broadly classified into two categories based on the severity of symptoms and serum Ca concentration. Mild hypocalcemia refers to a serum Ca level ranging between 1.9 to 2.1 mmol/L, which is usually asymptomatic or accompanied by mild symptoms. In contrast, severe hypocalcemia (SH) is defined by a serum Ca level less than 1.9 mmol/L, often associated with characteristic symptoms, and considered a medical emergency [6] with a potential risk of life-threatening arrhythmias or seizures [7]. Asymptomatic patients with a serum Ca level below 1.9 mmol/L may still experience severe complications, therefore requiring hospitalization for close observation and prompt treatment [8].

This study aimed to investigate the risk factors for SH after PTX in patients with PHPT and to analyze the predictive value of these clinical parameters. We hope to predict the risk of SH in patients with PHPT before surgery and take corresponding preventive measures, thus avoiding various dangerous clinical symptoms.

## Methods

### Patients

Between January 2010 and December 2022, Beijing Jishuitan Hospital in China conducted PTX on 214 patients. The inclusion criteria were mainly the diagnosis of PHPT, the indications for PTX and the success of PTX. Among the patients, 46 patients were subjected to rigorous data collection and comprehensive long-term follow-up. The patients successfully underwent PTX and were monitored from diagnosis to one-month post-operation. Prior to surgery, all patients received bisphosphonates for osteoporosis. Intravenous calcium supplementation started immediately after surgery with 10% calcium gluconate and normal saline restricted to a ratio of 1:1 and a speed of 50–100 ml/h. Calcium carbonate in conjunction with vitamin D were added after recovery of gastrointestinal function. The proportion of intravenous calcium supplementation was gradually reduced if serum Ca stabilized above 2.2 mmol/L. The diagnosis of PHPT was mainly based on atypical or elevated PTH levels and accompanying hypercalcemia. The Fourth International Workshop on the Management of Asymptomatic Primary Hyperparathyroidism guidelines were observed to determine surgical eligibility for both symptomatic patients (i.e., those with bone pain and/or urinary calculi and/or fragility fractures) and asymptomatic patients [9]. All patients underwent successful PTX with no evidence of residual disease or postoperative recurrence. The exclusion criteria were mainly the diagnosis of SHPT. Patients with normal parathyroid gland tissue (i.e., no hyperplasia, adenoma, or parathyroid cancer) as confirmed by histopathological examination following excision of the parathyroid lesions were excluded. Furthermore, patients were excluded from the study if they had a history of lithium or thiazide diuretic usage, or had undergone treatment with teriparatide, glucocorticoids, hormone replacement therapy, and/or had a diagnosis of familial hypocalcemia. The study was approved by the Ethics Committee of the Beijing Jishuitan Hospital.

### Clinical characteristics

The study's baseline characteristics encompassed demographic data, which consisted of variables such as sex, age, state of menstruation, family history, osteoporosis, urinary calculi, other endocrine diseases, and parathyroid pathology. Upon the specimens' removal, they underwent analysis, with professional pathologists conducting the pathological interpretation. Patients underwent biochemical evaluations two days before PTX and were followed at 1 h, 1 day, 3 days, 1 week and 1 month after surgery. Biochemical indicators were quantified using automated techniques, wherein serum Ca and serum phosphorus (serum P) levels were measured by an autoanalyzer (Hitachi H7600; Hitachi Corp. Tokyo, Japan). Intact parathyroid hormone (iPTH) levels were determined using a chemiluminescent immunometric assay (E601; Roche Diagnostics, Basel, Switzerland), while total procollagen type 1 intact N-terminal propeptide (tP1NP) and β type 1 cross linked C-terminal telopeptide collagen (βCTX) levels were assessed using commercial kits (Chemiluminescence, Elecsys 2010 analyzers; Roche Diagnostic, Indianapolis, IN, USA). Furthermore, osteocalcin (OC) and 25-hydroxyvitamin D3 levels were determined using a chemiluminescence assay (Roche, Elecsys 2010 analyzer, USA). Additional preoperative biochemical indicators included blood urea nitrogen (BUN), serum creatinine, serum uric acid, urine calcium (urine Ca), and urine phosphorus (urine P).

### Definition of postoperative SH

In this study, early postoperative SH was defined as a serum Ca concentration below 1.9 mmol/L within 48 h following surgery, even with the provision of standard supportive treatment. Notably, the cut-off value for serum Ca was consistent with that utilized in prior investigations, as reported in several studies [5–8]. It is also worth noting that patients without early postoperative SH had mild-to-moderate hypocalcemia or did not experience postoperative hypocalcemia because their postoperative serum Ca levels ranged from 1.9 to 2.3 mmol/L.

### Statistical analysis

Continuous data with a normal distribution are presented as mean ± standard deviation, while continuous data without a normal distribution are presented as medians (quartile 1, quartile 3). Categorical data are reported as frequencies. The student’s t-test or Mann–Whitney U test was used to compare continuous datasets, while the chi-square test or Fisher's exact test was used to compare categorical datasets. All considered factors underwent univariate analysis, and those with a *P* value below 0.05 on univariate analysis were included in a multivariate model to identify independent predictors. The strength of the association between each variable and the development of postoperative SH was evaluated using odds ratios (OR) and 95% confidence intervals (95% CI). Receiver operating characteristic (ROC) curve analysis was used to determine the optimal cut-off values for significant factors. All statistical analyses were conducted using IBM SPSS Statistics, version 22 (IBM Corp., Armonk, NY, USA, https://www.ibm.com/spss), and all tests were two-sided, with statistical significance defined as *P* < 0.05.

## Results

### Baseline characteristics of the patients

This study included 46 patients with confirmed PHPT who received PTX, as outlined in Table 1. The average age of the patients was 50.9 ± 15.5 years, and the majority of the participants were female (69.6%, *n* = 32). Of the female patients, 34.4% (*n* = 11) experienced menstruation. Of the entire group, 15 patients (32.6%) developed postoperative SH, 19 patients (41.3%) presented with urinary calculi, and 37 patients (80.4%) exhibited osteoporosis. Only one patient in the non-SH group had a family history of thyroid disease. Notably, there were no significant differences in sex, age, menstruation, urinary calculi, osteoporosis, or parathyroid pathological nature between the SH and non-SH groups.

**Table 1:** Comparison of demographic and laboratory data

Variable	All patients (*n* = 46)	SH group (*n* = 15)	Non-SH group (*n* = 31)	*P* value
Age(year, x ± s)	50.9 ± 15.5	45.3 ± 16.2	53.5 ± 14.8	0.094
Sex[n(%)]				1
Male	14(30.4%)	5	9	
Female	32(69.6%)	10	22	
Menstruation[n(%)]	11(34.4%)	4	7	0.96
Family history(n)	1	0	1	1
Endocrine disease(n)	4	0	4	0.288
Pathology[n(%)]				0.788
Adenoma	40(87.0%)	14	26	
Hyperplasia	3(6.5%)	1	2	
Cancer	3(6.5%)	0	3	
Calculi[n(%)]	19(41.3%)	7	12	0.607
Osteoporosis[n(%)]	37(80.4%)	12	25	1

*SH* group patients with severe hypocalcemia after parathyroidectomy, non-*SH* group patients without severe hypocalcemia after parathyroidectomy

### Comparison of laboratory parameters between patients with and without postoperative SH

The postoperative serum Ca levels of patients who developed postoperative SH were 1.77 ± 0.05 mmol/L, whereas the non-SH group had serum Ca levels of 2.07 ± 0.11 mmol/L, indicating a statistically significant difference (*P* < 0.001). The preoperative serum Ca (*P* = 0.012), serum Ca/P (*P* = 0.012), percentage decrease of serum Ca (*P* = 0.037), iPTH (*P* = 0.038), serum P (*P* < 0.001), tP1NP (*P* = 0.007), OC (*P* = 0.013), and ALP (*P* = 0.002) levels in the SH and non-SH groups were statistically significant (Table 2).

**Table 2:** Comparison of biochemical laboratory data

Variable	All patients (*n* = 46)	SH group (*n* = 15)	Non-SH group (*n* = 31)	*P* value
Postoperative serum Ca (mmol/L, x ± s)	1.97 ± 0.17	1.77 ± 0.05	2.07 ± 0.11	< 0.001*
Preoperative serum Ca (mmol/L, x ± s)	2.85 ± 0.32	2.71 ± 0.22	2.93 ± 0.34	0.012*
Decrease of serum Ca (mmol/L, x ± s)	0.88 ± 0.33	0.94 ± 0.23	0.85 ± 0.37	0.362
Percentage decrease of serum Ca (%, x ± s)	30.11 ± 9.03	34.07 ± 5.92	28.19 ± 9.71	0.037*
iPTH [pg/mL, M(Q1, Q3)]	461.7(174.8,887.7)	802.4(247.9,1282)	363(171.9,619.8)	0.038*
Serum P [mmol/L, M(Q1, Q3)]	0.66(0.57,0.81)	0.55(0.48,0.6)	0.72(0.59,0.88)	< 0.001*
Serum Ca/P (x ± s)	4.37 ± 1.20	5.00 ± 0.99	4.07 ± 1.19	0.012*
Urine Ca [mmol/24 h, M(Q1, Q3)]	3.77(2.99,5.30)	3.45(2.42,4.65)	3.92(3.15,5.61)	0.182
Urine P [mmol/24 h, M(Q1, Q3)]	9.94(6.37,13.36)	10.39(8.09,12.42)	9.42(6.08,13.54)	0.535
tP1NP [ng/mL, M(Q1, Q3)]	144.6(73.2,310.7)	257(146.8,549.3)	104.2(66.57,251)	0.007*
βCTX [ng/mL, M(Q1, Q3)]	1.30(0.59,1.97)	1.82(0.89,2.29)	1.01(0.57,1.82)	0.185
OC [ng/mL, M(Q1, Q3)]	106.5(33.3,161.9)	156.5(96.7,238)	58.2(29.9,141.3)	0.013*
25-(OH)-VD3 [ng/mL, M(Q1, Q3)]	9.02(6.9,13.7)	8.5(5.44,12.18)	9.71(7.91,14.6)	0.156
ALP [U/L, M(Q1, Q3)]	167(86,332)	296(209,553)	117(80,231)	0.002*
Serum creatinine [μmol/L, M(Q1, Q3)]	52(42,64)	45(35,63)	55(43,76)	0.125
BUN [mmol/L, M(Q1, Q3)]	4.05(3.2,5.225)	4(2.8,5.3)	4.1(3.6,5.2)	0.392
Serum uric acid (μmol/L, x ± s)	308.2 ± 89.1	314.53 ± 90.5	305.06 ± 89.71	0.739

*Serum Ca* serum calcium, *iPTH* intact parathyroid hormone, *serum P* serum phosphorus, *serum Ca/P* preoperative serum calcium/serum phosphorus, *urine Ca* urine calcium, *urine P* urine phosphorus, *tP1NP* total procollagen type 1 intact N-terminal propeptide, *βCTX* β type 1 cross linked C-terminal telopeptide collagen, *OC* Osteocalcin, *25-(OH)-VD3* 25-hydroxyvitamin D3, *ALP* Alkaline phosphatase, *BUN* Blood urea nitrogen

^*^ *P* < 0.05. SH group, patients with severe hypocalcemia after parathyroidectomy; non-SH group, patients without severe hypocalcemia after parathyroidectomy

### Relations of serum Ca after PTX and preoperative laboratory parameters

After PTX, serum Ca levels were significantly correlated with the preoperative levels of iPTH (correlation coefficient [*r*] = -0.44, *P* = 0.002), tP1NP (*r* = -0.57, *P* < 0.001), OC (*r* = -0.525, *P* < 0.001), ALP (*r* = -0.606, *P* < 0.001), serum P (*r* = 0.62, *P* < 0.001), preoperative serum Ca/P (*r* = -0.43, *P* = 0.003) and percentage decrease of serum Ca (*r* = -0.498, *P* < 0.001) (Table 3).

**Table 3:** Spearman correlation analysis for statistically significant variables and postoperative serum Ca

	Preoperative serum Ca	Percentage	iPTH	Serum P	Serum Ca/P	tP1NP	OC	ALP
*r*	0.14	-0.498	-0.44	0.62	-0.43	-0.57	-0.525	-0.606
*P*	0.351	< 0.001	0.002	< 0.001	0.003	< 0.001	< 0.001	< 0.001

*Serum Ca* Serum calcium, *r* correlation coefficient, *Percentage* Percentage decrease of serum Ca, *iPTH* intact parathyroid hormone, *serum P* serum phosphorus, *serum Ca/P* preoperative serum calcium/serum phosphorus, *tP1NP* total procollagen type 1 intact N-terminal propeptide, *OC* Osteocalcin, *ALP* Alkaline phosphatase

### Predictors of postoperative SH after PTX for PHPT

In this study, the preoperative levels of serum Ca, serum P, serum Ca/P, percentage decrease of serum Ca, ALP, tP1NP, and OC were considered as independent variables in the univariate regression analysis, with a predefined significance level of 0.05. The parameters that showed significant associations were then subjected to multivariate logistic regression analysis as presented in Table 4. The results indicated that four variables, namely the preoperative serum Ca level (OR = 0.007; 95% CI = 0.001–0.415; *P* = 0.017), serum P level (OR = 0.989; 95% CI = 0.981–0.996; *P* = 0.003), serum Ca/P (OR = 0.135; 95% CI = 0.019–0.947; *P* = 0.044) and the OC level (OR = 1.012; 95% CI = 1.001–1.024; *P* = 0.036), remained significantly associated with the occurrence of postoperative SH.

**Table 4:** Binary logistic regression analysis for the development of SH after PTX

Variable	Univariate			Multivariate		
	OR	95% CI	*P* value	OR	95% CI	*P* value
iPTH (pg/mL)	1.001	0.999 ~ 1.002	0.062			
Serum P (μmol/L)	0.989	0.982 ~ 0.997	0.006*	0.989	0.981 ~ 0.996	0.003*
Serum Ca (mmol/L)	0.069	0.005 ~ 0.880	0.04*	0.007	0.001 ~ 0.415	0.017*
Serum Ca/P	2.241	1.127 ~ 4.455	0.021*	0.135	0.019 ~ 0.947	0.044*
Percentage decrease of serum Ca (%)	1.091	1.002 ~ 1.189	0.045*	1.055	0.944 ~ 1.179	0.343
tP1NP (ng/mL)	1.005	1.001 ~ 1.008	0.013*	1.004	0.999 ~ 1.009	0.066
OC (ng/mL)	1.012	1.003 ~ 1.021	0.01*	1.012	1.001 ~ 1.024	0.036*
ALP (U/L)	1.004	1.001 ~ 1.007	0.019*	1.003	0.999 ~ 1.007	0.12

^*^ *P* < 0.05. *OR* Odds ratio, *95% CI* 95% confidence interval, *SH* Severe hypocalcemia, *PTX* Parathyroidectomy, iPTH intact parathyroid hormone, *serum P* serum phosphorus, *serum Ca* preoperative serum calcium, *serum Ca/P* preoperative serum calcium/serum phosphorus, *tP1NP* total procollagen type 1 intact N-terminal propeptide, *OC* Osteocalcin, *ALP* Alkaline phosphatase

### ROC curves for the serum P, serum Ca/P and ALP levels

The serum P, serum Ca/P and OC levels were independent predictors of postoperative SH. ROC curves were used to assess the potential application for early diagnosis of postoperative SH (Table 5). Based on the ROC curves, the cut-off values of serum P, serum Ca/P and OC were 0.575 mmol/L, 4.69 and 126.6 ng/mL, respectively. The sensitivity of serum P was 77.3%, specificity was 90.3%, and the area under the curve (AUC) was 0.859 (95% CI = 0.735–0.983). The sensitivity of serum Ca/P was 73.3%, specificity was 71%, and AUC was 0.735 (95% CI = 0.583–0.888). The sensitivity of OC was 73.3%, specificity was 74.2%, and AUC was 0.729 (95% CI = 0.563–0.895).

**Table 5:** ROC curve analysis for the prediction of postoperative SH

Variable	AUC (95% CI)	*P* value	Cut-off value	Sensitivity	Specificity
Serum P (mmol/L)	0.859(0.735 ~ 0.983)	< 0.001*	0.575	77.3%	90.3%
Serum Ca (mmol/L)	0.676(0.518 ~ 0.834)	0.55	3.08	38.7%	100%
Serum Ca/P	0.735(0.583 ~ 0.888)	0.010*	4.69	73.3%	71%
OC (ng/mL)	0.729(0.563 ~ 0.895)	0.013*	126.6	73.3%	74.2%


*ROC* Receiver operating characteristic, *SH* Severe hypocalcemia, *serum P* serum phosphorus, *serum Ca*, preoperative serum calcium, *serum Ca/P* preoperative serum calcium/serum phosphorus, *OC* Osteocalcin, *AUC* Area under the curve, *95% CI* 95% confidence interval

## Discussion

The clinical diagnostic evaluation of PHPT typically includes an assessment of various parameters such as serum Ca, iPTH, 24-h urine Ca, and serum 25-hydroxyvitamin D3. Additionally, treatment decisions may be influenced by evaluating bone density, bone turnover markers, ultrasound examination, radionuclide imaging, and CT scan results [10]. PTX is the definitive treatment for PHPT with the aim of resection of the parathyroid adenoma and biochemical cure of PHPT [2]. However, hypocalcemia is a common complication after PTX, often attributed to an acute reversal of bone calcium mobilization [3, 4]. The occurrence of postoperative hungry bone syndrome necessitates a drastic reduction in PTH release, which disturbs the balance of bone calcium efflux and influx during bone remodeling. In the high bone turnover state associated with hyperparathyroidism, PTH increases both bone formation and resorption. However, after PTX, the sudden decrease in PTH levels disrupts the balance between osteoblast-mediated bone formation and osteoclast-mediated bone resorption, leading to a significant net uptake of calcium, phosphorus, and magnesium by bone tissue [11].

Many studies on patients with hyperparathyroidism after PTX showed that preoperative iPTH [12–24], ALP [12–16, 18–20, 22–28], serum Ca [13–15, 17–28], serum P [12, 16, 22], and age [14, 18–23, 26] are influencing factors for postoperative SH. In the present study, there was a statistically significant difference in ALP and iPTH levels between the two groups, and both had a strong negative correlation with postoperative serum Ca levels, but there was no statistical significance in the binary logistic regression multivariate analysis. We believe that this may be due to the small sample size. We reported a notably high percentage of SH (32.6%) and it may be due to selection bias and small sample size. The introduction in clinical practice of the most recent guidelines on the management of mild primary hyperparathyroidism that suggest surgical removal also in case of mild presentation of the disease [9]. Our data were sourced from a wide range of time spans, with a small number of patients with mild PHPT in the past due to regional reasons, and a small number of patients who followed the new guidelines and were included in the study, which may have contributed to the bias.

In our study, all of the 3 cases of parathyroid carcinoma in the entire series were registered in the non-SH group. The preoperative serum Ca levels of the 3 cases were 2.68, 3.12 and 3.20 mmol/L which were relatively higher. We considered that preoperative serum Ca levels were higher in patients with parathyroid carcinoma and since it usually decreases slowly in postoperative days, in these patients the postoperative serum Ca level may be still sufficiently high (> 1.9 mmol/L) just after surgery.

Zhao S et al. reported that the volume of resected parathyroid glands was positively correlated with postoperative SH [14]. Specifically, a larger volume of resected parathyroid glands was associated with a greater incidence of postoperative hypoparathyroidism, as well as a lower secretion of PTH. This finding suggests that the likelihood of developing SH is higher in patients with a larger volume of resected parathyroid glands, as the decrease in PTH secretion makes them more susceptible to the condition. Wei et al., in a separate study [25], compared the recovery time from hypocalcemia between patients who underwent ultrasound-guided microwave ablation and those who underwent PTX. The authors found a statistically significant difference in the recovery time between the two groups, with the ultrasound-guided microwave ablation group requiring a shorter time to recover. This indicates that PTX has a relatively greater impact on parathyroid gland function, and patients who undergo this procedure may require a longer recovery time. In situations where it is feasible, patients may benefit from undergoing ultrasound-guided microwave ablation to mitigate the impact of long-term postoperative hypocalcemia. Cao et al., reported that parathyroid calcification may serve as a practical radiological predictor of postoperative SH. Specifically, their study revealed that the presence of parathyroid calcification was associated with a higher risk of SH in the short-term after surgery. This finding suggests that the identification of parathyroid calcification through radiological imaging may be a useful means of predicting the likelihood of developing SH following parathyroid surgery [13].

Liu et al. reported that the incidence of postoperative SH within 48 h was lower in the preventive calcium supplementation group compared to that in the routine calcium supplementation group [16]. As such, appropriate calcium supplementation within a range that does not induce hypercalcemia can be provided to patients with a high risk of postoperative SH in clinical practice, to prevent its occurrence. Yang et al. [29] have also conducted a study on the factors that affect the average decline rate of postoperative serum Ca before and after calcium supplementation. Preoperative ALP, iPTH, and hemoglobin levels were identified as independent factors affecting the decline rate of serum Ca, as well as the duration and dose of intravenous calcium supplementation. In addition, preoperative ALP and iPTH levels were identified as independent factors affecting the dose of oral calcium supplementation. Therefore, clinical practice should not only focus on the absolute decline in postoperative serum Ca levels but also pay attention to the decline rate of serum Ca and the duration of intravenous calcium supplementation to ensure adequate postoperative recovery. Ge et al. [30] reported that there was a significant correlation between the postoperative calcium supplementation demand and the preoperative ALP and iPTH levels. Their findings were consistent with those of other studies, suggesting that patients with risk factors, such as high preoperative ALP and iPTH levels, may require more perioperative management to prevent postoperative hypocalcemia. It is noteworthy that postoperative hypocalcemia was described in terms of postoperative treatment rather than symptoms, which is more relevant in clinical practice. In contrast to secondary hyperparathyroidism characterized by hypocalcemia and hyperphosphatemia, PHPT is typically identified by hypercalcemia, hypophosphatemia, and hypercalciuria. Notably, relatively low preoperative serum P levels may be indicative of higher bone resorption and mineral uptake after PTX [31, 32]. Consequently, it is essential to focus on the prevention and treatment of postoperative SH for patients with PHPT who exhibit typical hypophosphatemia.

Bone turnover markers are a set of biomarkers consisting of various proteins or protein derivatives that are released by osteoblasts or osteoclasts during bone remodeling. They have been shown to provide prognostic information on fracture risk, which complements radiographic measures of bone mass [33]. One such marker is OC, which is a serum protein that reflects osteoblast activity. Zou et al. [17] reported in a univariate analysis that OC values were found to be statistically significant between SH and normocalcemia groups. This study has identified OC as a potential risk factor for postoperative SH in patients with PHPT. The results reveal a relationship between bone turnover markers and postoperative SH. Specifically, a high preoperative OC level indicates a high state of bone remodeling. After PTX, the parathyroid function is inhibited, and the level of PTH drops sharply. This disruption in the balance between bone resorption and formation leads to a high rate of bone formation, which results in the rapid deposition of calcium salts into the bone tissue. This can cause hypocalcemia or even SH.

The primary focus of this study was to investigate patients with PHPT and identify new risk factors for postoperative SH after PTX. Specifically, the study highlights serum Ca/P and OC as novel risk factors for SH in patients with PHPT following PTX. Previous research has suggested that preoperative hyperphosphatemia is a risk factor for SH in patients with secondary hyperparathyroidism [18]. However, this study found that preoperative hypophosphatemia was a risk factor for SH in patients with PHPT. Reto Martin Kaderli et al. reported that the significant risk factor for hungry bone syndrome in multivariable analysis was high levels of preoperative iPTH [34]. We also recommend a consistent postoperative calcium and vitamin D supplementation to improve the bone metabolism.

It is important to note that this study has several limitations. Firstly, as a retrospective study, there was no recording of whether patients had symptoms associated with definite SH. Additionally, this was a single-center study with a relatively small sample size, which highlights the need for larger multicenter studies to verify these findings. It is also worth noting that differences in surgical skills and experience among surgeons performing PTX may affect postoperative recovery of parathyroid function. Finally, the empirical use of postoperative intravenous calcium supplementation may potentially mask underlying SH in patients.

In summary, this study identified several risk factors for postoperative SH in patients with PHPT who underwent PTX. Specifically, the preoperative low serum P level, high serum Ca/P and OC levels were found to be significant risk factors for developing SH after surgery. Therefore, it is recommended that patients who exhibit these risk factors in clinical practice should be closely monitored for serum Ca levels after surgery and be supplemented with calcium as necessary. Bruno Madeo et al. reported that the serum Ca/P ratio is a highly accurate index to identify PHPT when Ca/P is above 2.55 [35]. These results demonstrate the reliability of this index to rule in/out primary parathyroid dysfunctions and remark the importance of measuring serum P in clinical practice. While these findings provide valuable insights, it is important to note that more clinical data should be collected to further investigate these factors. Moreover, clinical trials are necessary to validate the influence of these factors for prospective research. Future research should aim to expand on the current findings and further elucidate the mechanisms underlying the development of SH in patients with PHPT after PTX.

## Conclusion

Preoperative serum P, serum Ca/P and OC levels may identify patients with PHPT at risk for early postoperative SH after PTX.

## Data Availability

The data that support the findings of this study are available from the corresponding author upon reasonable request.

## Abbreviations

PHPT: Primary hyperparathyroidism
SH: Severe hypocalcemia
PTX: Parathyroidectomy
serum P: Serum phosphorus
OC: Osteocalcin
OR: Odds ratio
95% CI: 95% Confidence interval
AUC: Area under the curve
PTH: Parathyroid hormone
serum Ca: Serum calcium
iPTH: Intact parathyroid hormone
tP1NP: Total procollagen type 1 intact N-terminal propeptide
βCTX: β Type 1 cross linked C-terminal telopeptide collagen
BUN: Blood urea nitrogen
urine Ca: Urine calcium
urine P: Urine phosphorus
ROC: Receiver operating characteristic
